# Supplementary material for: Unveiling the Bioactive Potential of the Invasive Jellyfish Phyllorhiza punctata Through Integrative Transcriptomic and Proteomic Analyses
Source: Biomolecules. 2025 Aug 4;15(8):1121. doi: 10.3390/biom15081121 (PMC12383608; doi:10.3390/biom15081121)
Supplement: Supplementary file 1 [file biomolecules-15-01121-s001.zip › Suplementary Material/FileS3/PEPFOLD4/XP_065055784_702/clusters.html]

 PEP-FOLD clusters


Download archive of all models  here   

|  |  |  |
| --- | --- | --- |
| # | file | sOPEP |
| # cluster 1 - Size: 50 |  |  |
|  | XP\_065055784\_702-model1 | -13.8479 |
|  | XP\_065055784\_702-model1.1 | -13.8479 |
|  | XP\_065055784\_702-model1.2 | -13.8479 |
|  | XP\_065055784\_702-model1.3 | -13.8479 |
|  | XP\_065055784\_702-model1.4 | -13.8479 |
|  | XP\_065055784\_702-model1.5 | -13.8479 |
|  | XP\_065055784\_702-model1.6 | -13.8479 |
|  | XP\_065055784\_702-model1.7 | -13.8479 |
|  | XP\_065055784\_702-model1.8 | -13.8479 |
|  | XP\_065055784\_702-model1.9 | -13.8479 |
|  | XP\_065055784\_702-model1.10 | -13.8479 |
|  | XP\_065055784\_702-model1.11 | -13.8479 |
|  | XP\_065055784\_702-model1.12 | -13.8479 |
|  | XP\_065055784\_702-model1.13 | -13.8479 |
|  | XP\_065055784\_702-model1.14 | -13.8479 |
|  | XP\_065055784\_702-model1.15 | -13.8479 |
|  | XP\_065055784\_702-model1.16 | -13.8479 |
|  | XP\_065055784\_702-model1.17 | -13.8479 |
|  | XP\_065055784\_702-model1.18 | -13.8479 |
|  | XP\_065055784\_702-model1.19 | -13.8479 |
|  | XP\_065055784\_702-model1.20 | -13.8479 |
|  | XP\_065055784\_702-model1.21 | -13.8479 |
|  | XP\_065055784\_702-model1.22 | -13.8479 |
|  | XP\_065055784\_702-model1.23 | -13.8479 |
|  | XP\_065055784\_702-model1.24 | -13.8479 |
|  | XP\_065055784\_702-model1.25 | -13.8479 |
|  | XP\_065055784\_702-model1.26 | -13.8479 |
|  | XP\_065055784\_702-model1.27 | -13.8479 |
|  | XP\_065055784\_702-model1.28 | -13.8479 |
|  | XP\_065055784\_702-model1.29 | -13.7297 |
|  | XP\_065055784\_702-model1.30 | -13.7297 |
|  | XP\_065055784\_702-model1.31 | -13.7297 |
|  | XP\_065055784\_702-model1.32 | -13.7297 |
|  | XP\_065055784\_702-model1.33 | -13.7297 |
|  | XP\_065055784\_702-model1.34 | -13.7297 |
|  | XP\_065055784\_702-model1.35 | -13.7297 |
|  | XP\_065055784\_702-model1.36 | -13.7297 |
|  | XP\_065055784\_702-model1.37 | -13.665 |
|  | XP\_065055784\_702-model1.38 | -13.665 |
|  | XP\_065055784\_702-model1.39 | -13.665 |
|  | XP\_065055784\_702-model1.40 | -13.5164 |
|  | XP\_065055784\_702-model1.41 | -13.5164 |
|  | XP\_065055784\_702-model1.42 | -13.5164 |
|  | XP\_065055784\_702-model1.43 | -13.5164 |
|  | XP\_065055784\_702-model1.44 | -13.5164 |
|  | XP\_065055784\_702-model1.45 | -13.5164 |
|  | XP\_065055784\_702-model1.46 | -13.3619 |
|  | XP\_065055784\_702-model1.47 | -13.3619 |
|  | XP\_065055784\_702-model1.48 | -13.3391 |
|  | XP\_065055784\_702-model1.49 | -13.1002 |
| # cluster 2 - Size: 1 |  |  |
|  | XP\_065055784\_702-model2 | -10.5243 |
| # cluster 3 - Size: 2 |  |  |
|  | XP\_065055784\_702-model3 | -9.84281 |
|  | XP\_065055784\_702-model3.1 | -9.21132 |
| # cluster 4 - Size: 5 |  |  |
|  | XP\_065055784\_702-model4 | -9.205 |
|  | XP\_065055784\_702-model4.1 | -9.06958 |
|  | XP\_065055784\_702-model4.2 | -8.90355 |
|  | XP\_065055784\_702-model4.3 | -8.87162 |
|  | XP\_065055784\_702-model4.4 | -8.70983 |
| # cluster 5 - Size: 36 |  |  |
|  | XP\_065055784\_702-model5 | -9.20194 |
|  | XP\_065055784\_702-model5.1 | -9.10908 |
|  | XP\_065055784\_702-model5.2 | -9.1018 |
|  | XP\_065055784\_702-model5.3 | -9.1018 |
|  | XP\_065055784\_702-model5.4 | -9.00829 |
|  | XP\_065055784\_702-model5.5 | -9.00829 |
|  | XP\_065055784\_702-model5.6 | -8.98437 |
|  | XP\_065055784\_702-model5.7 | -8.98437 |
|  | XP\_065055784\_702-model5.8 | -8.96427 |
|  | XP\_065055784\_702-model5.9 | -8.96427 |
|  | XP\_065055784\_702-model5.10 | -8.96427 |
|  | XP\_065055784\_702-model5.11 | -8.96427 |
|  | XP\_065055784\_702-model5.12 | -8.94832 |
|  | XP\_065055784\_702-model5.13 | -8.88676 |
|  | XP\_065055784\_702-model5.14 | -8.88676 |
|  | XP\_065055784\_702-model5.15 | -8.86528 |
|  | XP\_065055784\_702-model5.16 | -8.85989 |
|  | XP\_065055784\_702-model5.17 | -8.85989 |
|  | XP\_065055784\_702-model5.18 | -8.85545 |
|  | XP\_065055784\_702-model5.19 | -8.79162 |
|  | XP\_065055784\_702-model5.20 | -8.77064 |
|  | XP\_065055784\_702-model5.21 | -8.74663 |
|  | XP\_065055784\_702-model5.22 | -8.73319 |
|  | XP\_065055784\_702-model5.23 | -8.7271 |
|  | XP\_065055784\_702-model5.24 | -8.7271 |
|  | XP\_065055784\_702-model5.25 | -8.69895 |
|  | XP\_065055784\_702-model5.26 | -8.68733 |
|  | XP\_065055784\_702-model5.27 | -8.6684 |
|  | XP\_065055784\_702-model5.28 | -8.65135 |
|  | XP\_065055784\_702-model5.29 | -8.61263 |
|  | XP\_065055784\_702-model5.30 | -8.60233 |
|  | XP\_065055784\_702-model5.31 | -8.57466 |
|  | XP\_065055784\_702-model5.32 | -8.57466 |
|  | XP\_065055784\_702-model5.33 | -8.55038 |
|  | XP\_065055784\_702-model5.34 | -8.54282 |
|  | XP\_065055784\_702-model5.35 | -8.47276 |
| # cluster 6 - Size: 5 |  |  |
|  | XP\_065055784\_702-model6 | -9.1277 |
|  | XP\_065055784\_702-model6.1 | -8.95398 |
|  | XP\_065055784\_702-model6.2 | -8.66105 |
|  | XP\_065055784\_702-model6.3 | -8.66105 |
|  | XP\_065055784\_702-model6.4 | -8.59186 |
| # cluster 7 - Size: 1 |  |  |
|  | XP\_065055784\_702-model7 | -8.98042 |
|  |  |  |
